# Toxin-linked mobile genetic elements in major enteric bacterial pathogens

**DOI:** 10.1017/gmb.2023.2

**Published:** 2023-03-17

**Authors:** Shruti Panwar, Shashi Kumari, Jyoti Verma, Susmita Bakshi, Lekshmi Narendrakumar, Deepjyoti Paul, Bhabatosh Das

**Affiliations:** Functional Genomics Laboratory, Infection and Immunology Division, Translational Health Science and Technology Institute, Faridabad, India

**Keywords:** Mobile genetic elements, microbiome, pathogens, toxins, horizontal gene transfer, drug resistance

## Abstract

One of the fascinating outcomes of human microbiome studies adopting multi-omics technology is its ability to decipher millions of microbial encoded functions in the most complex and crowded microbial ecosystem, including the human gastrointestinal (GI) tract without cultivating the microbes. It is well established that several functions that modulate the human metabolism, nutrient assimilation, immunity, infections, disease severity and therapeutic efficacy of drugs are mostly of microbial origins. In addition, these microbial functions are dynamic and can disseminate between microbial taxa residing in the same ecosystem or other microbial ecosystems through horizontal gene transfer. For clinicians and researchers alike, understanding the toxins, virulence factors and drug resistance traits encoded by the microbes associated with the human body is of utmost importance. Nevertheless, when such traits are genetically linked with mobile genetic elements (MGEs) that make them transmissible, it creates an additional burden to public health. This review mainly focuses on the functions of gut commensals and the dynamics and crosstalk between commensal and pathogenic bacteria in the gut. Also, the review summarises the plethora of MGEs linked with virulence genes present in the genomes of various enteric bacterial pathogens, which are transmissible among other pathogens and commensals.

## Introduction

**Figure 1:**
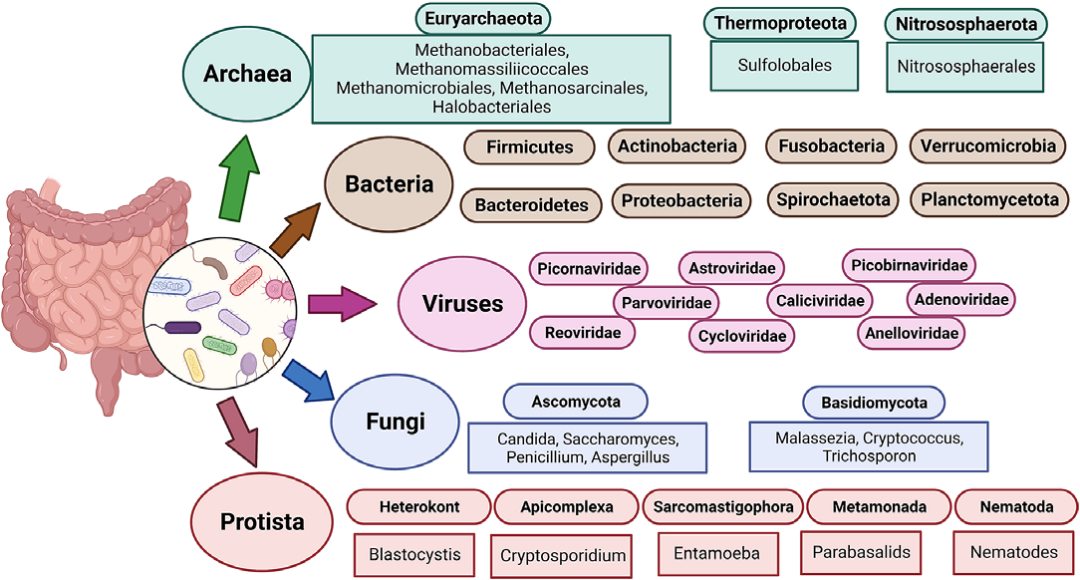
The diverse human gut microbiota. Population of Archaea, Bacteria, Fungi and Protists are part of the complex ecosystem of the human gut microbiota. The graphic shows the diverse compositions that dominate in different domains of life.

The term “gut microbiota” refers to the whole population of microbes that live in the human gut and includes bacteria, fungi, archaea, protozoans and viruses (Sekirov et al., 2010). Humans’ bacterial microbiota has been thoroughly studied, but research on the other kingdoms is still in its early stages. An overview of the diverse human gut microbiota’s constituents is shown in Figure 1 along with its predominant members. The gut has a large supply of micronutrients, a wide pH range and access to oxygen, hydrogen and methane, hence making it a preferred location for microbial colonisation and a suitable niche for horizontal gene transfer (HGT) (Kurokawa et al., 2007). Over the past 10 years, a number of studies have highlighted the interactions between bacteria and their hosts. Within the human gut, bacteria can be either commensal, symbiotic, pathobiont or pathogenic (Matijašić et al., 2020). The gut commensals play important roles in vitamin production, short-chain fatty acids (SCFA) synthesis, barrier function regulation, immunomodulation and many more, thus supporting the body’s homeostasis (Valdes et al., 2018). They are known to inhibit the growth of pathogenic bacteria through a process known as “colonisation resistance,” either by producing metabolites that inhibit the pathogen growth or by regulating the host immune system. However, there are also a few reports that suggest commensals may aid pathogen’s colonisation by secreting nutrients that feed these bacteria, allowing them to eventually outcompete the commensals and cause disease (Rolhion and Chassaing, 2016). Additionally, previous research has shown that commensals produce small compounds from the host mucin layer that modulate the virulence of enterohaemorrhagic *Escherichia coli* (Jubelin et al., 2018). It has been well reported that pathogenic microbes have the ability to exchange genes with the non-pathogenic residents of the gut (Messerer et al., 2017). To comprehend how infections evolve in the gut, one must have a thorough understanding of the ecology and genetic characteristics of the many bacteria that predominate in the human gut. The development of methods and technologies over the past 20 years, particularly the introduction of next-generation sequencing, has improved our capacity to comprehend and examine the contributions of the microbiota members and their roles in human health.

**Figure 2:**
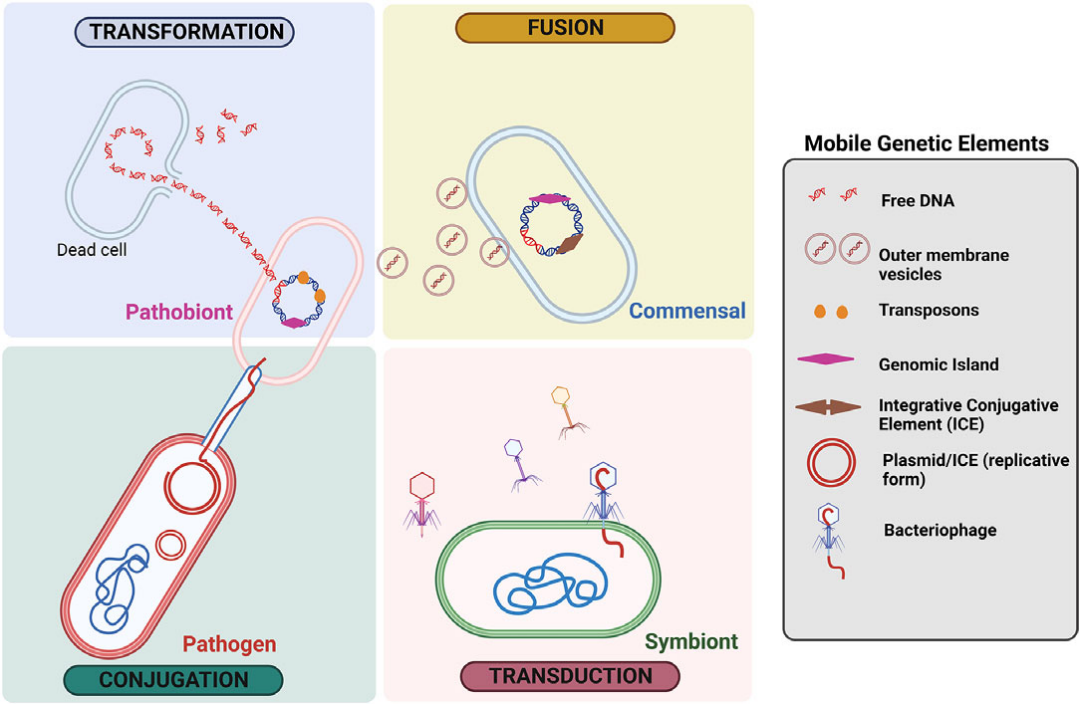
The mechanisms of gene exchange in human gut microbiota. The known mechanisms for mediating horizontal gene transfer (HGT) include transformation, transduction, conjugation and the fusion of outer membrane vesicles. Antibiotic resistance genes, virulence and pathogenicity determinants are transmitted by various mobile genetic elements (MGEs) through HGT. The widespread HGT in the human gut microbiome has a significant impact on both health and disease.

A bacterial genome is divided into two broad categories, such as the core and the accessory genome. The accessory genome is a growing gene pool with non-essential capabilities that provide an advantage for survival in a given niche, whereas the core genome encodes proteins that are required for metabolic functions (Rankin et al., 2011). Antibiotic-resistant (Acman et al., 2022) and virulence genes (Juhas et al., 2015) are among the gene pools found in the accessory genome, and they are mostly spread by horizontal transfer processes (Brito et al., 2016), that is, transduction, conjugation, natural transformation and fusion of outer membrane vesicles (Figure 2). Compared to the core genome, the exogenic DNA is easily distinguishable due to its unique G + C composition and specific insertion sites (Ochman et al., 2000). Mobile genetic elements (MGEs) are one of the major facilitators of HGT (Gyles and Boerlin, 2013). MGEs include prophages, composite transposons, pathogenic islands, phages, integrative conjugative elements (ICEs), plasmids, etc. (Davis and Waldor, 2002). The transfer of MGEs within the gut results in the transmission of not only antibiotic resistance genes (ARGs) but also genes that code for metabolic competences such as bile salt detoxification, polysaccharide utilisation and mucus degradation (Broaders et al., 2013). The richness and diversity of these MGEs in the human gut make it difficult to fully understand their ecological and biological identities. Many published articles have already established that the MGEs are acquired via HGT and linked with antibiotic resistance coding genes (Kent et al., 2020; von Wintersdorff et al., 2016; Wang et al., 2022), but the MGEs linked to virulence genes are less highlighted (Partridge et al., 2018).

Numerous bacterial chromosomes and mobile genetic components have toxin–antitoxin (TA) systems (Peltier et al., 2020; Schmidt and Hensel, 2004; Weaver et al., 2017). Commensals usually lack virulence features, and they actively produce substances that promote stable interactions with other bacteria that prevent their entry into potentially harmful pathways. Changes in ecologies that create new habitats or the transmission of virulence genes from pathogens lead to the transformation of commensal to pathogenic. Acquisition of toxins or genes linked to disease, such as pathogenicity islands, are examples of mechanisms that contribute to the transformation of commensals into pathogens and the destabilisation of the commensal/host interaction (Gilmore et al., 2013). Alternatively, loss of commensal functions can lead to virulence, as appears to have happened in the cases of *Yersinia pestis* (Chain et al., 2004) and *Bordetella pertussis* (Parkhill et al., 2003). The present review summarises the different MGEs present in the genomes of enteric pathogens and other commensal bacteria present in the gut and their roles in toxin production, pathogenesis and disease development. This comprehensive review sheds light on the role of MGEs in shaping the ecology and evolution of the gut microbiome and how they result in community adaptations to the gut environment.

## The dynamic human gut microbiome

**Figure 3:**
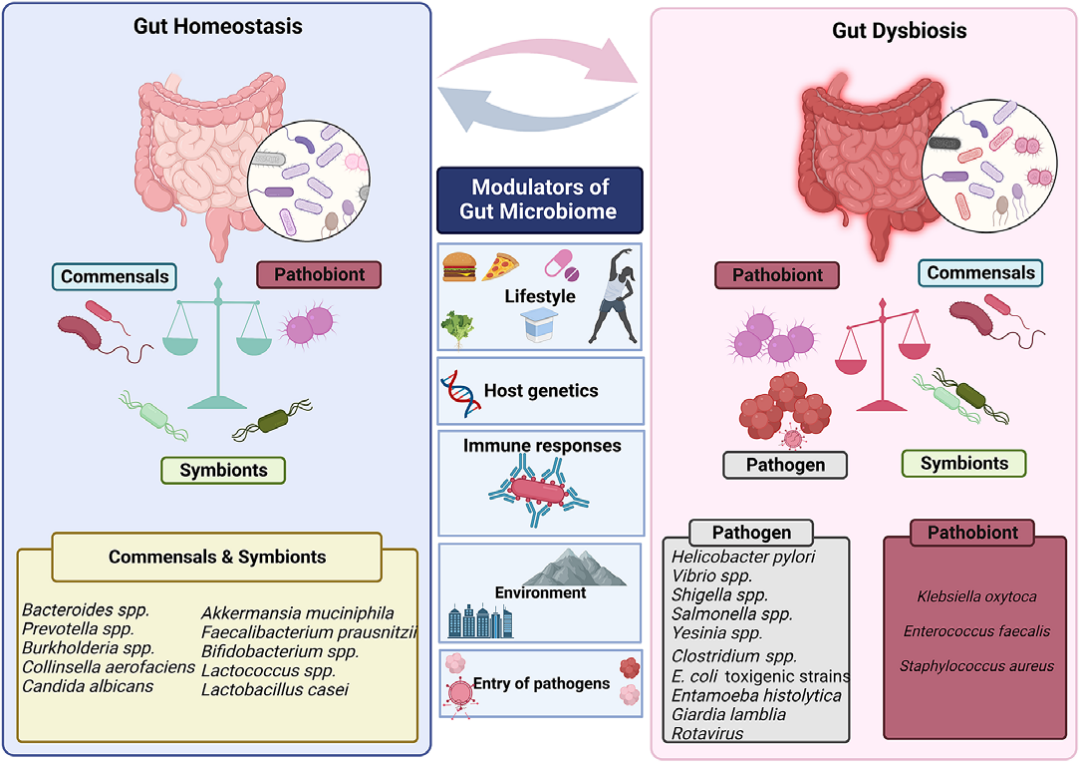
Factors that modulate gut’s microbial ecosystem. Numerous variables including lifestyle, age, genetics of the host, environment, pathogen infiltration, immune responses and so forth result in dynamic changes that may put the gut microbiota in a dysbiotic state. The dysbiosis of the gut microbiota leads to change in the abundance of commensals and symbionts, which is associated with a diverse range of human illnesses and disorders.

Human gut is an abode to a complex and dynamic microbial community. A wide range of variables, including the delivery method at birth (Reyman et al., 2019), diet (David et al., 2014; Muegge et al., 2011), lifestyle and host genetics (Qin et al., 2022) influence the composition of the gut microbiota. Evolutionary dynamics like mutation, HGT, drift and selection, as well as ecological factors like changes in species abundance or strain replacements, influence the gut microbiome (Garud and Pollard 2020). However, even today, a major gap exists in our knowledge of the global microbiome variability. It has been established that industrialisation, westernisation and the rural–urban divide within a nation are the main causes of this heterogeneity (De Filipo et al., 2017). Environmental factors, genetics, food, illnesses and antibiotic exposure all play a significant role in determining the diversity and composition of microorganisms in various body locations. The diverse range of factors that can affect gut homeostasis and microbial diversity is depicted in Figure 3.

The five major phyla of gut bacteria are Firmicutes, Bacteroidetes, Actinobacteria, Proteobacteria, Fusobacteria and Verrucomicrobia, with Firmicutes and Bacteroidetes accounting for 90 per cent of the gut microbiota of a healthy human being (Arumugam et al., 2011). The Firmicutes phylum is composed of more than 200 different genera composing *Lactobacillus*, *Bacillus*, *Clostridium*, *Enterococcus* and *Ruminococcus.* *Clostridium* genera represent 95 per cent of the Firmicutes phyla. Bacteroidetes consist of predominant genera such as *Bacteroides* and *Prevotella.* The Actinobacteria phylum is proportionally less abundant and mainly represented by the *Bifidobacterium* genus. The major gut pathogens are *Bacteroides fragilis*, *Clostridium perfringens*, *Clostridium botulinum*, *C. difficile*, *Enterococcus faecalis*, *Staphylococcus aureus*, *Salmonella* sp., *Shigella* sp., *Vibrio parahaemolyticus*, *V. cholerae*, *Yersinia* sp. and *Helicobacter pylori* belonging to Bacteroidetes, Firmicutes and Proteobacteria phyla. The gut microbiota also differs according to the anatomical areas of the intestine, which also have different physiological characteristics, pH and oxygen tension, substrate abundance and host secretions (Zhang et al., 2015).

Due to the dynamic nature of the gut microbiome, there are significant differences in the composition and diversity of the gut microbiome among people of different nations. Some bacteria are specific to people of a particular geographical location. Further, specific genes of bacteria have also been identified to be solely present in people of a specific geographical location or ethnic group. According to research performed by Chen and colleagues (Chen et al., 2020), higher abundance of the *ppsA* gene was only observed in *Pseudomonas stutzeri* of the European population. Further, *Burkholderia pseudomallei* S13 is known to be more widespread in the European population. Additionally, it has been found that the prevalence of Bacteroides is higher in European and American populations than in Asian people, and the gene MH0053_GL0075770 has been associated with fat metabolism and could be correlated with the high-fat diet of European and American populations (Chen et al., 2016). Furthermore, research has shown that *Prevotella* and *Treponema* are more prevalent in people of Burkina Faso, an African country whose people strictly adhere to a vegetarian diet (De Filippo et al., 2010). Interestingly, children in Japan were identified to possess a unique microbiome with high prevalence of *Bifidobacteriaceae* and low presence of *Enterobacteriaceae*, highlighting the highly hygienic lifestyle of the Japanese population and their eating habits (Nakayama et al., 2015). The prevalence of *Bacteroides plebeius* in the gut microbiome of Japanese population which can metabolise porphyran present in seaweeds establishes the link between diet and GI microbiome (Hehemann et al., 2010).

According to Das et al. (2018), Firmicutes, Bacteroidetes, Actinobacteria and Proteobacteria dominate the gut microbiome in Indian communities. *Prevotella* and *Candida* were more prevalent in Indians than in Japanese because of the plant-based diet of the Indian population (Pareek et al., 2019). Another intriguing study by Rothschild et al. (2018) supports the idea that environmental factors dominate in the formation of the gut microbiome. Individual single nucleotide polymorphisms (SNPs) or genetic ancestry do not significantly influence the microbiota, and previously reported relationships are not consistently observed across investigations (). Although environmental influences are thought to be the main element influencing the development of the gut microbiome, individual genetics also have a role in microbiome composition (Jakobsson et al., 2010). Several genome-wide association studies have linked host genetic variations in immunity-related pathways to microbiome composition in healthy and diseased conditions (Blekhman et al., 2015). Mutations in the Mediterranean fever gene (MEFV) were found to be associated with changes in the gut microbiome community structure (Khachatryan et al., 2008). Various host genetic factors and host immune factors identified to have a role in shaping human microbiome are listed in the “Host Genetic and Immune factors shaping human Microbiota (GIMICA)” database (Tang et al., 2021). Further, differential exposure to a variety of antibiotics also alters a person’s microbial profile. Observational studies have found a negative relationship between the prevalence of microbial communities and antibiotic exposure (Korpela et al., 2016). Interestingly, non-antibiotic drugs like metformin used majorly to treat Type 2 diabetes were also identified to cause dysbiosis of commensal bacteria within the gut (Forslund et al., 2015). The review discusses the functions of commensal bacteria in the gut, the major gut pathogens and dynamics of MGEs between the commensals and pathogens that are important in gut homeostasis and disease progression.

## Functions of commensal bacteria in the gut

The presence of commensal bacteria in the gut is known to maintain gut homeostasis and have a significant impact on human health and disease. Kyoto Encyclopedia of Genes and Genomes (Kanehisa and Goto, 2000) analysis of 1520 Culturable Genome Reference of commensal bacteria revealed that they were more involved in carbohydrate and amino acid metabolism (Zou et al., 2019). The phyla Fusobacteria, Bacteroidetes, Proteobacteria and other Gram-negative bacteria were identified to possess a wide range of lipopolysaccharide biosynthesis genes (ko00540). Genes that function towards the glycan degradation (ko00531 and ko00511) were identified to be abundant in Bacteroidetes suggesting its involvement in carbohydrate catabolism. Further, genes involved in sphingolipid metabolism (ko00600) and steroid hormone synthesis (ko00140) were identified to be abundant in Bacteroidetes. Proteobacteria were identified to be rich in genes involved in xenobiotic degradation (ko01220) (Zou et al., 2019). However, many virulence factors and ARGs were also mapped by virulence factor database (Chen et al., 2005) and Comprehensive Antibiotic Resistance Database (Alcock et al., 2020) in bacteria belonging to the Proteobacteria phylum suggesting its ability to cause diseases.

Essential coenzymes like cobalamin are captured by commensals in the gut using surface-exposed lipoproteins (Wexler et al., 2018). Biosynthesis of queuosine, a substitute for guanine, having relevance in many physiological defects like cancer progression, neurological deformities and increased cell proliferation was identified to be performed by *E. coli* and *Bacillus subtilis.* This was established by studying a queuosine biosynthesis gene mutant *E. coli* which accumulated epoxyqueuosine (Miles et al., 2011). The main products of the saccharolytic fermentation of carbohydrates, known as SCFA, are formate, acetate, propionate and butyrate, which have a variety of functions in maintaining healthy intestinal physiology, including barrier integrity, immunomodulation, epithelium proliferation and appetite regulation (Chambers et al., 2014; Magne et al., 2020; Morrison and Preston, 2016).

It has been understood that intestinal commensals breakdown dietary fibres to release indole derivatives, which activates AhR (aryl hydrocarbon receptor) and initiates ILC3 (type 3 innate lymphoid cells) cells to strengthen intestinal mucosa by interleukin-22 (Postler and Ghosh, 2017). Further, intestinal commensal bacteria metabolise arginine to secrete polyamines which inhibit NLRP6 (NOD-like receptor family pyrin domain containing 6) inflammasome and also alleviate pro-inflammatory cytokines (Levy et al., 2015). Gut microbiome metabolites are also known to inhibit nuclear factor-κB-dependent synthesis of pro-inflammatory genes that modulates cytokines (Zhang et al., 2022). Additionally, it is known that gut bacteria can alter bile salts generated by the host, which are important for signalling and increasing epithelial barrier function (Sayin et al., 2013). Polysaccharide A synthesised by *B. fragilis* acts as an anti-inflammatory molecule which induces the secretion of IL-10 by CD4+ T cells (Johnson et al., 2015). It has been determined that *Clostridia* maintains the level of retinoic acid in the gut by inhibiting the activity of retinol dehydrogenase 7 (Rdh7) in intestinal epithelial cells (Grizotte-Lake et al., 2018). Thus, gut commensals play a significant role in modulating the host health by various methods such as nutrient metabolism, drug clearance, barrier integrity maintenance and immunomodulation. Bifidobacteria and *Lactobacillus* spp. are also widely used as probiotics in the nutraceutical industry, and certain species have a long history of safe use in the manufacture of food, feed and effectiveness in rejuvenating dysbiotic gut due to infection or antibiotic use. However, their use is jeopardised by the erythromycin resistance gene erm(X) translocation, which is mediated by the genomic island BKGI1 (Li et al., 2022).

## Pathogenic bacteria in the gut

Every year, GI tract infections kill millions of people worldwide. Since bacteria are the most common cause of GI illnesses, antibiotics are frequently used to treat them. The use of antibiotics results in intestinal dysbiosis and, in extreme situations, sepsis due to the release of antibiotic-induced endotoxins (Lepper et al., 2002). *Escherichia*, *Salmonella*, *Shigella*, *Vibrio*, *Yersinia*, belonging to the phyla Proteobacteria, and *Clostridia*, belonging to Firmicutes, are some common genera of enteric pathogens. These bacterial pathogens have been identified to possess several toxin genes that have been found to be linked to MGEs that could facilitate its transfer to opportunistic pathogens and commensal bacteria of the gut. The toxin genes of enteric pathogens and their associations with various MGEs that can aid in the transfer of these toxin genes have been detailed in the sections below.

### B. fragilis

*B. fragilis* is a rod-shaped, Gram-negative obligate anaerobe belonging to the phyla Bacteroidota. Genome of *B. fragilis* National Collection of Type Cultures (NCTC) 9343 is widely studied and harbours one single circular chromosome of 5205140 bp harbouring 4274 genes and a plasmid pBF9343 (Pierce and Bernstein, 2016). Although this bacterium is commensal in humans, a subset of it called Enterotoxigenic *B. fragilis* (ETBF) has been linked to major human illnesses such as colorectal cancer and inflammatory diarrhoea. When clinical isolates of ETBF were compared to the reference strain NCTC 9343, it was identified that the clinical isolates had 23 per cent acquired genes that were responsible for toxins and antibiotic resistance (Pierce and Bernstein, 2016). Pathogenic island was identified to have a reduced G + C content (35 per cent) as compared to the flanking DNA (47–50 per cent) suggesting that the ETBF isolates acquired the pathogenicity island through HGT from some other bacteria in the gut or from another pathogen during a transient infection. Additionally, the toxin gene *bft-2* and metalloprotease gene (*mpII*) (Moncrief et al., 1995) were identified to be flanked by putative mobilisation genes *bfmA, bfmB* and *bfmC*, and the BfPAI itself is flanked by a mobilisation region similar to that of the plasmid pIP417 known for 5-nitro-imdazole resistance and plasmid pBFTM10 known to provide clindamycin resistance (Haggoud et al., 1994). The proteins synthesised from the *bfmC* gene were identified to be similar to the *TraD* mobilisation protein of *E. coli* plasmid F and R100 (Franco Augusto et al., 1999). Also, the ETBF strains possess a 20 kDa metalloprotease toxin gene called fragilysin responsible for cytotoxicity of intestinal cells in the fragilysin pathogenicity islet present on a transposable element CTn*86.* Apart from CTn*86*, there are other putative conjugative transposons CTn*9343*, CTn*9343*-like or CTn*86*-like elements in the regions flanking the pathogenicity islands of ETBF (Buckwold et al., 2007).

### C. perfringens

*C. perfringens* is a spore forming, rod-shaped, Gram-positive anaerobe belonging to the Bacillota/Firmicutes phyla and is widely found in the gut of healthy humans. However, occasionally, *C. perfringens* causes various intestinal discomforts and enteric diseases like food poisoning, food independent diarrhoea and colitis (Uzal et al., 2010). Complete genome sequence of 56 enterotoxin-producing *C. perfringens* isolated from patients having food poisoning demonstrated that they possessed a diverse pangenome with only 12.6 per cent core genome suggesting the occurrence of high rate of HGT and acquisition of new genes that contribute to toxin production, antibiotic resistance and persistence (Kiu and Hall, 2018). *C. perfringens* type A strains were identified to possess a putative open reading frame (ORF) showing homology to an ORF of *Salmonella Typhimurium* IS*200* insertional element 1.5 kb upstream of *cpe* gene that codes for the *C. perfringens* enterotoxin responsible for the toxicosis (Brynestad et al., 1997). Further, it was identified that the epsilon toxin (*etx*) gene present in type B and D strains of *C. perfringens* is flanked by IS*1151* and a gene linked with Tn*3* transposon that shows similarity with the gene coding transposase in *S. aureus* and *Lactococcus* (Brynestad et al., 1997; Uzal et al., 2010). The IS*1151* located 96 bp upstream of the *etx* gene in *C. perfringens* type D strains was identified to be homologous to the insertion sequence (IS) elements of *Bacillus thuringiensis* and *E. coli* (Daube et al., 1993). Few *C. perfringens* type A strains were also identified to possess the *cpe* gene on a large plasmid that contained an IS*1470* element in its chromosome (Brynestad et al., 1994). The IS*1470* element carried the gene coding for a 346 aa transposase enzyme which showed homology with the transpose carried by IS*30* (Brynestad et al., 1994). Also, the genome of *C. perfringens* was identified to be rich in phage elements such as ϕSM101, ϕ3626, ϕS9, ϕS63, ϕCP26F, ϕCP390, ϕCPV4, ϕZP2, ϕCP7R, ϕCPV1 and ϕCP24R (Kim et al., 2012).

### C. botulinum

*C. botulinum* is a rod-shaped, motile, spore forming, Gram-positive anaerobe belonging to the Bacillota/Firmicutes phyla that produce neurotoxin botulinum. Human botulism is caused due to the consumption of contaminated food and can cause neurotoxicity and even paralysis in humans (Nigam and Nigam, 2010). There are four groups of *C. botulinum* of which group I and II cause botulism in humans. Group III causes botulism in animals and group IV has no association with botulism (Peck, 2009). According to a 2017 report, the complete genome of only 13 strains of *C. botulinum* was available at NCBI (http://www.ncbi.nlm.nih.gov/genbank/). However, in 2022, there are about 35 complete genome sequences and 440 partial genome sequences of *C. botulinum.* The genome size of *C. botulinum* ranged from 3.2 to 4.2 Mb with a GC content of 27–29 per cent (Bhardwaj and Somvanshi, 2017). The *bont* gene cluster encodes for the botulinum neurotoxin (BoNT) that inactivates acetylcholine anchors in neuromuscular junctions and causes paralysis. The presence of *bont* genes in *C. botulinum* is identified due to HGT. The *bont* gene cluster is either present in the chromosome or plasmid of the bacterium. In *C. botulinum* strain A ATCC 3502, *bont* genes are present within *oppA/brnQ* operon, *arsC* operon or *rarA* operon (Skarin and Segerman, 2011). Furthermore, BoNT is divided into types A, B, C, D, E, F and G. The group II *C. botulinum* is largely isolated from food-borne infections and is known to produce B, E and F neurotoxin. In *C. botulinum*, A, B and F toxins are chromosomally encoded, toxin G is encoded by plasmid and prophages encode C1, D and E (Brüssow et al., 2004; Skarin and Segerman, 2011). The *C. botulinum* G toxin was identified to be present on an 81 MDa plasmid, and *C. botulinum* type C strain (C)-203 U28 was identified to possess the C2 toxin on a large plasmid designated as pC2C203U28. Further, in-depth genomic analysis of *C. botulinum* revealed that group III strains possess a variety of other toxins encoded in plasmids (Nawrocki et al., 2018). A recent report suggests that the group I and II *C. botulinum* have many *bont* clusters flanked by IS elements, which allows the mobility of these genes within the genome and also could be transferred to other bacteria (Sakaguchi et al., 2009). Additionally, the C2 toxin genes were identified to be linked with IS elements like ISCbt5 and ISCbt6 (Sakaguchi et al., 2009). Though a large number of IS elements and plasmids have been identified in the *C. botulinum* genome, not much information is present on the prevalence of phage elements in the genome apart from those that harbours the *botC* and *D* genes (Hill et al., 2009). However, five phages c-st, c-468, c-203, c-d6f and d-1873 were identified to be responsible for converting non-toxigenic strains of *C. botulinum* type C and D to toxigenic strains (Sakaguchi et al., 2005). Additionally, infection of two bacteriophages, CEβ and Ceγ, was revealed to convert non-toxigenic strains to toxigenic (Eklund et al., 1971).

### C. difficile

*C. difficile* is an anaerobic, Gram-positive, rod-shaped bacterium belonging to phyla Bacillota, Firmicutes known to cause diarrhoeal disease and colitis in humans. There are more than 2600 genomes of *C. difficile* deposited in Genbank as of 2022. The complete pangenome of *C. difficile* was estimated to have around 9640 genes acquired mainly through HGT events which constitute around 11 per cent of the total genome (Eyre et al., 2013; Scaria et al., 2010). Many plasmids have been identified to possess genes that confer antibiotic resistance to *C. difficile.* Many studies have reported the presence of transposons that confer antibiotic resistance like Tn*5397* or CTn*3* (tetracycline resistance), Tn*5398* (macrolide–lincosamide–streptogramin resistance) in the past. Virulence factors of *C. difficile* are toxin A (clostridial cytotoxin) and B, encoded by *tcdA* and *tcdB* genes on a 19.6 kb long region of chromosome forming a distinct pathogenic locus (*PaLoc*). Further, *tcd*B and *cdt*AB that code for the binary toxin with ADP-ribosyltransferase activity were identified to be coded by putative conjugative plasmids. *C. difficile* Clade C-I strains were identified to carry a monotoxin *tcdB* ^+^ PaLoc next to a full CdtLoc on extrachromosomal molecules that resemble conjugative plasmids (Ramírez-Vargas and Rodríguez, 2020). Additionally, the PaLoc encodes proteins that regulate and help in the secretion of the toxin. The transfer of PaLoc was identified to convert a non-toxigenic strain to toxigenic (Brouwer et al., 2013). PaLoc is absent in non-toxic strains. A 115-bp DNA fragment was found between two ISs cdu 2/2′ and cdd 2–3 located upstream and downstream to PaLoc (Braun et al., 1996). While in other strains like VPI 10463 the toxigenic element is 19.6 kb in length and contains five ORFs. Four of these ORFs are toxin A, toxin B, ORFtxe2 and ORFtxe3 and ORFtxel (Hammond and Johnson, 1995). Interestingly, the exact mechanism of transfer of the PaLoc among the strains is not fully understood. Till date, not much data are available for the presence of transposons that are linked with the mobility of virulence or toxin genes in *C. difficile* (Brouwer et al., 2011). IStrons are a combination of group I intron and an IS which can splice out entirely and transpose to a new location. IStrons are capable of possessing variant proteins as they have the unique splicing activity. Insertion of IStron into the *C. difficile* toxin A has been found to be responsible for the bacterium to produce alternative variant toxins. Rupnik et al. (2016) studied the various permutations of toxins produced by the different toxin types of *C. difficile.*

### E. faecalis

*E. faecalis* is a Gram-positive, belonging to the phyla Bacillota, Firmicutes and is a natural resident of the GI tract of humans and is frequently observed in the faecal material. Though the bacteria is considered to be a commensal, it has also been associated with many nosocomial (healthcare-associated) infections including urinary tract infections, bacteremia, wound infections and endocarditis (Fowler et al., 2005; Murray, 1990; Tleyjeh et al., 2005). The reference strain of *E. faecalis* V583, a clinical isolate, was first reported, sequenced and published in 2003 in the USA. It contained 3337 ORFs that encode for proteins in its chromosome and three plasmids pTEF1, pTEF2 and pTEF3. Chromosomal G + C content of the strain was 37.5 per cent, whereas plasmids revealed a G + C content of 33.3–34.4 per cent and encoded for 3240 proteins. A total of the 25 per cent of *E. faecalis* genome mainly consist of several MGEs such as 38 insertional elements, 7 phage regions, pathogenicity islands and regions for composite transposable elements. Majority of the MGEs were identified to carry ARGs and virulence genes (Giridhara Upadhyaya et al., 2009; Paulsen et al., 2003). In *E. faecalis*, the virulence factors mainly include the adherence, biofilm formation, quorum sensing and the toxin genes. Adherence factors such as the *ebpA/B/C* (pili aiding in bacterial adherence to host proteins), *ace* (collagen adhesin) and *asa1* (aggregation substance) were associated with the virulence of the organism (Fiore et al., 2019). The toxin cytolysin of *E. faecalis* was identified to be produced by the genes present in the *cyl* operon (toxin cytolysin) which comprises 8 genes *cylA/B/I/M/R_1_/R_2_/S* (Fiore et al., 2019). Additionally, few strains of *E. faecalis* were identified to produce bacteriocins, which is encoded by a conjugative plasmid pMB1 of 90 kb in size and responsive to sex pheromones released by other bacteria that facilitate its transfer (Martínez-Bueno et al., 1992).

Previous studies have unveiled that the most virulent strains of *E. faecalis* are MDR and strong biofilm formers since they get an upper hand in surviving in the gut as compared to other susceptible enteric bacteria (Mundy et al., 2000). The *esp* gene-encoded enterococcal surface protein (Esp) is responsible for the biofilm formation that allows its colonisation in the GI tract (Kristich Christopher et al., 2004). Clinical strains of *E. faecalis* were observed to contain pathogenicity islands that harboured both cytolysin and *esp* when compared with non-infective oral-derived isolates (Gold et al., 1975). Isolates were also identified to harbour prophage-like elements which are mostly associated with virulence and pathogenicity. Strain V583 contains seven prophage-like elements which fall under the category of temperate phages V583-pp1 to V583-pp7 with size ranging from 12 to 43 Kb (Matos et al., 2013). Apart from the temperate phages, lysogenic phages were also reported, namely GQ478081 (ΦFL1A), GQ478082 (ΦFL1B), GQ478083 (ΦFL1C), GQ478084 (ΦFL2A), GQ478085 (ΦFL2B), GQ478086 (ΦFL3A), GQ478087 (ΦFL3B) and GQ478088 (ΦFL4A) (Stevens et al., 2011). Phage DNA integrates into the host bacteria via integrase belonging to the serine recombinase family at *att* sites in the chromosome. Proteins encoded from the gene of the phages are either involved in lysogeny maintenance, adhesion and virulence (Brede et al., 2011).

### S. aureus

*S. aureus* is a Gram-positive bacterium, again in the phyla Bacillota, Firmicutes and an opportunistic pathogen that colonises different parts of the human body. However, the bacterium is also known to cause diseases like food poisoning, toxic shock syndrome, pneumonia, sepsis and endocarditis. *S. aureus* is a major contributing cause for the hospital-acquired infections and is notoriously known for acquiring virulence genes encoded by MGEs (Lindsay and Holden, 2004). The genome of *S. aureus* ranges from 2.8 Mb to 2.9 Mb. About 75 per cent of the *S. aureus* genome was identified to be conserved which forms the core genome and is involved in regular metabolism of the cell. About 25 per cent of the genome was identified to be an accessory genome that contained a lower G + C content as juxtaposed to the core genome (Turner et al., 2019).

Like in other bacteria, genes associated with virulence and pathogenicity comprise the accessory genome. *S. aureus* isolates contain one or more plasmids naturally and are classified into three classes, I, II and III. It was identified that in *S. aureus* most plasmid transfer occurs through transduction as *S. aureus* is not conjugatively competent. Many ARGs of *S. aureus* have been associated with plasmids. *van A* operon that contains genes that confer resistance against vancomycin is understood to be attained by *E. faecalis* as a result of conjugal transfer (Hiramatsu et al., 1997). Apart from the genes that codes for vancomycin resistance, genes that code for resistance against beta-lactam antibiotics were also identified to be present in the plasmids of *S. aureus* (Altboum et al., 1985). Additionally, enterotoxin B, bacteriocin and exfoliative toxin B were identified to be plasmid encoded in the pathogen (Bukowski et al., 2010). Six genes (*seg*, *sei*, *sem*, *sen*, *seo* and *seu*) encoding enterotoxins are located on the enterotoxin gene cluster (*egc*), which is part of the *S. aureus* genomic island *v*Saβ (also known as SaPI3/m3). The transfer of *v*Saβ is facilitated by Staphylococcal temperate phage, *Φ* SaBov (Moon et al., 2015).

Apart from plasmids, genetic elements like transposons and IS elements that aid in the bacterial evolution were also identified to be present in *S. aureus* genome in single or tandem copies. ISs and unit transposons are also known to greatly contribute to antibiotic resistance in *S aureus* (Byrne et al., 1989). Apart from antibiotic resistance, the transposons also confer resistance to heavy metals like cadmium (Kuroda et al., 2001). Phage elements of *S. aureus* are of three types, lytic, temperate and chronic. Furthermore, based on the size of the phage element, it is divided into class I (16–20 kb), II (35–40 kb) and III (125–140 kb) (Kwan et al., 2005). In *S. aureus*, temperate bacteriophages contain genes like staphylokinase (*sak*), chemotaxis inhibition protein (*scn*), enterotoxins and exfoliative toxin (eta) (Deghorain and Van Melderen, 2012). Virulence factors such as Panton-Valentine leucocidin, enterotoxin A and exfoliative toxin A are encoded by lysogenic prophages. Virulence-associated genes are generally present near the attachment (*att)* site and integrative (*int)* site of the phage element. Helper phages *Φ*11 and *Φ* 80 α aid in the replication, mobilisation and excision of Staphylococcal pathogenicity islands (SaPI), which is a non-mobile pathogenic island of *S. aureus* (Mir-Sanchis et al., 2012; Ram et al., 2012). Many SaPIs have been sequenced, which encode enterotoxins and toxic shock syndrome toxin (TSST) (Xia and Wolz, 2014).

### Salmonella spp

*Salmonella* is an enterobacterial Gram-negative, rod-shaped bacteria that come under the phylum Pseudomonadota, that is, Proteobacteria. They are facultative anaerobes, which are responsible for a significant amount of disease burden globally. *Salmonella* spp. is known as one of the major causes of GI illness worldwide. Globally, 1.3 billion instances of gastroenteritis, 3 million fatalities and 16 million cases of typhoid fever are all attributed to *Salmonella* each year (Pui et al., 2011). *Salmonella enterica* and *Salmonella bongori* are two of the species that make up the genus *Salmonella.* More than 2600 serotypes of *S. enterica* are further split into six subspecies, and they are distinguished from one another by differences in their flagellar (H) and somatic (O) features. The majority of human infections are caused by *S. enterica* subspecies I (enterica), and it is also the most isolated subspecies in animals (Brenner et al., 2000). On the other hand, *S. bongori* has been found mostly in “cold-blooded” animals such as amphibians, fish and reptiles and is also known to cause less than 1 per cent of human infection (Tomastikova et al., 2017). Salmonellae are categorised medically into typhoidal (*S. Paratyphi* A*, S. Paratyphi* B*, S. Typhi*) and non-typhoidal *Salmonella* (e.g., *Enteritidis*). *S. Typhi* murium is known to cause typhoid fever, *S. Paratyphi* A, B and C cause enteric fever and other serotypes of *S. Paratyphi* cause salmonellosis. *Salmonella* serovars that are known to cause gastroenteritis can spread through contaminated food or water or directly through the faecal–oral route. The majority of *Salmonella* serotypes can cause gastroenteritis, whereas a small number, like *S. Typhi*, can result in an invasive infection (Rabsch et al., 2001). The pathogenicity of *Salmonella* infections involves a wide range of virulence factors such as Salmonella pathogenicity islands SPI-1, SPI-2 and other SPIs that are encoded with type 3 secretion systems (T3SS), as well as flagella, capsules, plasmids and adhesion systems. The development of a T3SS-2 and intracellular reproduction takes place in a membrane-bound compartment known as the *Salmonella*-containing vacuole (SCV). Two conserved and stable PAIs, known as Salmonella pathogenicity islands 1 and 2 (SPI-1 and SPI-2, respectively), are present in all *S. enterica* species. SPI-1 expressed a secretion system of type 3 (TTSS-1), containing invasion genes that enable the bacteria to enter its host intestinal epithelial cells via a process involving actin polymerisation and cytoskeleton remodeling (Raffatellu et al., 2005, Jajere 2019). Furthermore, SPI-2, a TTSS-2 encoder, is synthesised when Salmonella infects host phagocytic cells such as dendritic cells and macrophages which facilitates the survivability of *Salmonella* in the vacuole known as a “SCV” by delaying the development of the vacuole and its fusion with lysosomes. *Salmonella* proliferation in conditions with low magnesium levels such as in the macrophages depends on SPI-3 (Amavisit et al., 2003, Foley et al., 2013). Genes located on the SPI-4 are necessary for intra-macrophage survival, apoptosis and the release of toxins. SPI-5 genes encode a variety of T3SS effector proteins, while genes encoded by SPI-6 transport proteins into the cellular environment or host cells in response to external stimuli. Moreover, *S. enterica* subsp. enterica possessed a large excisable PAI, Salmonella pathogenicity island 7 (SPI-7) containing around 150 genes. The SPI-7 is about 134 kb in size and has a GC content of approximately 49.7 per cent. SPI-7 was identified to be highly mosaic and appears to have been derived by sequential acquisition of different genes. The pathogenicity island apart from possessing genes that are involved in its mobilisation has also been identified to harbour virulence genes such as the Vi antigen, SopE phage and a type IVB pilus locus (Bueno et al., 2004). The *sopE* virulence gene (STY4609) encodes SopE protein, an effector protein released by the TTSS-1 that causes actin rearrangement in epithelial cells was identified to be a part of a P2-like prophage located in the middle of SPI-7. *S. enterica* serovar Enteritidis (*S. enteritidis*) is a pathogenic bacterium which possesses an unstable pathogenicity island of 26.5 kb named Region of Difference 21 or ROD21 (SPI19). The ROD21, pathogenicity island was identified to be present in the chromosome of *S. enteritidis* linked to a number of virulence genes (Pardo-Roa et al., 2019). Salmonella and various distinct serotypes have been discovered to contain temperature-dependent, diversified and host-limited IncC, IncF, IncHI and IncI1 conjugative plasmids, comprising AR genes. In particular, the IncF conjugative virulence plasmid was acquired from an avian pathogenic *E. coli* strain (Lindsey et al., 2009).

### Vibrio parahaemolyticus

*Vibrio parahaemolyticus* is a Gram-negative, curved, rod-shaped, halophilic bacterium belonging to the phyla Pseudomonadota, Proteobacteria that causes food-borne GI illness in humans on the consumption of improperly cooked seafood (Daniels et al., 2000). *V. parahaemolyticus* was first discovered in 1950 after an outbreak of seafood poisoning in Japan (International Symposium on *Vibrio parahaemolyticus, 1974*). Additionally, *V. parahaemolyticus* has been linked to cause septicaemia and wound infections in humans (Santos et al., 2020). Apart from infections in humans, the pathogen also causes infection in shrimp [acute hepatopancreatic necrosis disease (AHPND)], which is an emerging disease, initially named as early mortality syndrome (Tena et al., 2010). AHPND is not only caused by *V. parahaemolyticus* but also caused by other members of *Vibrio* sp. such as *V. campbellii*, *V. owensii* and *V. punensis.* Interestingly, it has been identified that pVA1-type plasmid carries the *pirAB^vp^* toxin gene responsible for the disease. Further, it was identified that the plasmid can be transferred among the Vibrio spp. through conjugation. The pVA1-type plasmid was identified to have a GC content of roughly 45.9 per cent with a copy number of 37 per bacterial cell, and it comprised of 92 ORFs that encode virulence-associated proteins, mobilisation proteins, replication enzymes, transposases and other proteins which are related to the toxins from the Photorhabdus insect-related (Pir) toxins (Lee et al., 2015). Two genes, *pirA*- and *pirB*-like, which are located within a 3.5 kb fragment region, are flanked by 1 kb inverted repeats transposon-coding sequence and are associated for encoding Pir toxin-like proteins in *V. parahaemolyticus.* The GC content of these 2 genes was found to be substantially lower (38.2 per cent) than the remainder of the plasmid, which suggests that these genes have been acquired through horizontal transfer. *V. parahaemolyticus* and *V. cholerae*, the cholera-causing agent, share a phylogenetic relationship. They both have two circular chromosomes. *V. parahaemolyticus* genome has two chromosomes, which are about 3288558 bp and 1877212 bp and possess 4832 genes, with a G + C content of 45·4 per cent for each chromosome. The chromosome I of both *V. parahaemolyticus* and *V cholerae* is identified as not much different in size (3·3 vs 3·0 Mb), but the chromosome II of *V. parahaemolyticus* was identified to be larger in size than that of *V. cholerae* (1·9 vs 1·1 Mb) (Tagomori et al., 2002). There are several plasmids identified in *V. parahaemolyticus* such as pSA19, pZY5 and p22702B. Most of the genes in these plasmids were known to encode hypothetical proteins. The studies on ICEs of *V. parahaemolyticus* are sparse; however, in 2019, a study by He et al. (2019) identified ICE positive *V. parahaemolyticus* isolated from aquacultured shrimp. The ICE was reported to harbour mainly genes that code for antibiotic resistance and heavy metal resistance. In contrast to the limited studies of *V. parahaemolyticus* plasmids and ICEs, there have been numerous studies on the phage elements that have been acquired by the pathogen and its contribution to its pathogenicity. There have been reports of filamentous vibriophages such as the f237 identified from O3:K6 pandemic clones of *V. parahaemolyticus.* Other well-characterised phage elements in *V. parahaemolyticus* include KVP40, VP882, VP93, pO3K6, Vf12, Vf33, VfO3K6, VfO4K68 and VpV262. There has been significant amino acid similarity identified among the *V. parahaemolyticus* filamentous phages and the phages identified from other species of the *Vibrionaceae* family (Chang et al, 1998). Additionally, there has been evidence of other HGT events in the *V. parahaemolyticus* genome. There has been high similarity observed in the T3SS located on chromosome II of *V. parahaemolyticus* and non-O1/non-O139 *V. cholerae* strains. The second T3SS2 of *V. parahaemolyticus* located on chromosome II was identified to harbour two copies of *tdh* (thermostable direct haemolysin) flanked by Tn*7*-like transposase genes. Further, evidence suggests that the *V. parahaemolyticus* acquired the *trh* (TDH-related haemolysin) from *V. alginolyticus* in an event of HGT (González-Escalona et al, 2006; Xie et al, 2005). HGT has been identified to cause emergence of pathogenic clones of *V. parahaemolyticus* from the environment.

### H. pylori

*H. pylori* is a microaerophilic Gram-negative, helical bacteria belonging to the phyla Campylobacterota, Proteobacteria. This bacterium is present in the mucus that colonises the stomach’s epithelium in more than 50 per cent of the world’s population (Bravo et al., 2018; Proença-Modena et al., 2009). The disease severity mainly depends upon both the host factors and the bacterial factors. Most of the time, the infection is asymptomatic, but occasionally it can develop into peptic ulcers, mucosa-associated lymphoid tissue lymphoma and even stomach cancer (GC). In 1994, *H. pylori* was categorised by the World Health Organization as a class I carcinogen (“Schistosomes, liver flukes and *Helicobacter pylori* (1994). IARC Working Group on the Evaluation of Carcinogenic Risks to Humans. Lyon, 7–14 June 1994,” ). *H. pylori* are spiral, rod-shaped, curved bacteria having flagella and a membrane sheath outer covering. Motility is another crucial virulence component of their pathogenicity that allows the bacteria to pass through the mucin layer of the gastric epithelium (Josenhans and Suerbaum, 2002). Once the bacterium attaches to the gastric epithelial cells, it causes vacuolation of the epithelial cells resulting in cell injury. This vacuolation is the result of the production of a cytotoxin called vacuolating cytotoxin A (VacA), a pore-forming, secreted toxin that is responsible for causing extensive vacuolation in epithelial cells, cell death and epithelial integrity disruption (Szabò et al., 1999). Vacuolisation may differ significantly from strain to strain, and there has been correlation between the severity of *H. pylori* pathogenesis and the existence of a cytotoxin-associated gene pathogenicity island (PAI). An important virulence factor is the cagA that is present within an island of approximately 30 genes, most probably acquired by *H. pylori* from other organisms. The clinically important *H. pylori* has been divided into type I and type II strains. All type I strains have genes that can make both the cytotoxins CagA and VacA, while type II strains only have genes which are necessary that can make VacA. *H. pylori* has a quite complicated pathophysiology. There are several MGEs in the genome of *H. pylori*, and several studies have reported that there has been genetic rearrangement within the genome of the pathogen that helps it adapt to the harsh gastric condition and also express virulence and resistance genes. A recent study reported the ICEs of *H. pylori* type four secretion system (ICE*Hptfs*) are a conserved genomic area in *H. pylori.* Though the region was identified to be conserved, it was reported to be able to mobilise via conjugation. Additionally, the region portrayed high allele diversity. The ICE element was identified to harbour genes that code for the type 4 secretory system (T4SS), *VirB*, *D* and *C* genes. Apart from the ICEs in the genome of *H. pylori*, the pathogen is also known to possess cryptic plasmids which provide regions that are hot spots for site specific recombination. Interestingly, the pathogen is also identified to possess plasmids that reveal homology to those of Gram-positive organisms which replicate via rolling circle mechanism and also possess plasmids that replicate via the theta mechanism. Additionally, there have been several IS elements identified in *H. pylori* that harbours genes that show homology to the genes of other pathogens such as *Salmonella* (virulence gene *gipA*) and *E. coli* (Vale et al., 2008).

### Other enteric pathogens

It has been estimated that half of all the diarrhoeal diseases are due to enteric Gram-negative bacteria. They contribute a significant portion of the burden of diarrhoea and enteric fever which cause more than three million fatalities annually. The major cause of the diarrhoeal infection is the production of one or more bacterial enterotoxins. Other important gut pathogens belonging to the phylum Proteobacteria, Pseudomonadota are *V. cholerae* and *E. coli.* *V. cholerae* have been associated with one of the most severe diarrhoeal infections, cholera, while infections caused by Enterotoxigenic *E. coli* (ETEC) are responsible for the greatest number of traveller’s diarrhoea. The other important GI diarrhoeal diseases caused by enteric pathogens include Shigella spp., which belongs to the phyla Pseudomonadota, Proteobacteria and *Campylobacter jejuni* that belongs to Campylobacterota, Proteobacteria. Among viruses, rotavirus is known to cause the most severe diarrhoeal illness among kids under the age of 2–3. Caliciviruses and several adenovirus varieties are further significant GI viruses. Parasitic enteric pathogens also cause diarrhoeal cases that include *Entamoeba histolytica*, *Giardia lamblia* and Cryptosporidium spp. These pathogens cause infections by different methods. In general, the conventional infectious cycle includes (1) entry of the pathogen, (2) the establishment and growth of pathogens inside the host cell, (3) evasion of host defences and (4) damage to host and exit. Majority of these functions are achieved by the enteric pathogens with the help of a diverse array of effector molecules. The effector molecules broadly can be classified as those that help the bacterium in the colonisation and establishment of the pathogen in the host gut and the others that help the pathogen for transmission which is achieved by damaging the host cells. The pathogen also produces effector molecules that help the pathogen to evade host immunity.

The enterotoxin that the ETEC strains produce is similar to the cholera toxin (CT), and both cholera and ETEC diarrhoea result in large amounts of water and electrolytes, secreted by the small intestine’s upper fifth. ETEC infection requires adhesion initially and then followed by the synthesis of toxins. ETEC produces two varieties of enterotoxins, a 84-kd heat-labile toxin (LT) and the other ETEC toxin is heat stable (ST) STa and STb. ST has a temperature tolerance of 100 °C and only STa, a peptide with a size of around 2 kD, has been linked to human disease (Joffré et al., 2016). Both human and swine genomes have a wide range of genes that encode for various LT variations. Heat-labile enterotoxin (LT) variants LTIp, LTIh, LTIc and LTIIa, encoded by the eltAB gene, have reportedly been related to plasmids, chromosomes and prophages (Jobling et al., 2012, 2016; Lasaro et al., 2008), while the majority of heat-stable toxin variants in humans and pigs have been related to plasmids (Joffré et al., 2016; Taillon et al., 2008). Both ETEC and *V. cholerae* have comparable fimbriae, which are crucial for bacterial adhesion and colonisation in the host’s small intestine. Colonisation factors, which are encoded on plasmids, play an essential role in mediating adhesion, *tia* an outer membrane adhesin molecule is another important virulence factor, encoded within a pathogenicity island (Fleckenstein, et al., 1996). In addition to the contrasts, there are similarities. The fluid secretion in *cholera* is largely, though not exclusively caused by a single enterotoxin. But the LT (heat-labile toxin) and ST (heat-stable toxin) enterotoxins are the one(s) or both that induce acute toxicity-related diarrhoeal disease. CT genes are encoded by a prophage (CT phage) located chromosomally whereas in case of ETEC, both the ST and LT genes are found on plasmids and are not phage associated. Majority of the GI pathogens, including the EPEC, *Salmonella Shigella* and *Yersinia* use its T3SS to deliver the effector proteins into the host cells. *Shigella* readily invades the epithelial cells of the human intestine from the basolateral surface. The *Shigella* sp. contains a single circular chromosome and a virulence plasmid. The virulence plasmid has been associated with the virulence and pathogenesis of the pathogen. Majority of the virulence factors of *Shigella* are situated in a 30 kb region termed as the “entry region” which contains mxi-spa locus, which encodes a T3SS. This large plasmid also encodes for the proteins (IpaB and IpaC) that help the bacteria to enter the host cells, multiply and spread to adjacent cells (Sansonetti et al., 1999). In addition to the virulence plasmid, pathogenicity islands (PAI) on the *Shigella* chromosome also harbour genes that contribute to the virulence of the pathogen. Interestingly, it has been identified that the genes and other elements in the PAI can be found in a variety of combinations depending on the *Shigella* species and subtype. A combination of both chromosomal virulence factors and plasmid virulence factors mediate the invasiveness and virulence of the pathogen. *Shigella* enterotoxin 1 (ShET1) and *Shigella* enterotoxin 2 (ShET2) are major virulence factors for mediating early fluid secretion in the jejunum and then subsequently in the colon. ShET1 is encoded by *set1A* and *set1B* genes on the *Shigella* chromosome as part of the SHI-1 PAI. The PAI is specific to only *S. flexneri* 2a isolates (Vargas et al., 1999; Yavzori et al., 2002). The two toxin subunits together form the holo-AB-type toxin complex in an A1-B5 configuration, similar to that of the cholera holotoxin, and are secreted via Sec pathway and Type II secretion (Faherty et al., 2012).

Another major enteric pathogen is the *Yersinia* again a member of phyla Pseudomonadota, Proteobacteria, and three species, namely *Y. pestis*, *Y. enterocolitica* and *Y. pseudotuberculosis*, are known to cause lethal disease in humans. The pathogen is associated with causing infection in regional lymph nodes or lungs and also a broad range of GI diseases, from enteritis to mesenteric lymphadenitis (Bibikova, 1977; Putzker et al., 2001; (Pujol and Bliska, 2005). Virulent *Yersinia* species have several virulence factors, like a 70-kb virulence plasmid, pCD1 in *Y. pestis* and pYV in enteropathogenic *Yersinia.* They also encode for the yersiniabactin (Ybt) system (Brubaker, 1991; Cornelis et al., 1998; Heesemann et al., 1993). The 70-kb virulence plasmid in *Y. pestis* has been identified to harbour several genes that code for the structural components of a T3SS, and also the T3SS effector proteins called *Yersinia* outer proteins (Yops) (Bliska et al., 2013; Schwiesow et al., 2015). The Yops protein is known to help the pathogen in immune evasion. *Yersinia* species also possess a number of T6SSs with distinct biological functions. The T6SSs delivers multiple effector proteins while other secretory systems are known to deliver a single type of effector protein. In addition to effector proteins that are toxins, some effector molecules delivered via the T6SS system also enhance the persistence of the pathogen. The T6SS is also identified to have a role in the biofilm formation of a bacteria (Southey-Pillig et al., 2005). The different toxin genes associated with MGEs in different bacterial enteric pathogens have been summarised in Table 1.

**Table 1.** Mobile genetic elements associated with toxin genes in bacterial pathogens causing enteric diseases.

Bacterial Phyla	Organism	Disease	Toxin	Toxin genes	Associated MGEs	References
*Bacteroidetes*	*Bacteroides fragilis*	Modifies the surface proteins and junctional proteins	BFT 1,2,3 proteins	*bft-1*, *bft-2* and *bft-3*	Pathogenicity islands (BfPAI), CTn86 phage	Scotto d’Abusco et al. (2000)
Proteobacteria	Enterotoxigenic *Escherichia coli* (ETEC)	Gastroenteritis, diarrhoea	Heat-stable toxin (ST) and heat-labile toxin (LT)	*estA*, *eltB*	Transposons, plasmid	Sjöling et al. (2006)
	Enteroaggregative *E. coli* (EAEC) *E. coli* O104:H4	Gastroenteritis, diarrhoea	Plasmid encoded toxin (Pet), heat-stable toxin (EAST1) and Shigella enterotoxin 1 (ShET1)	*astA*, *aatA*	Plasmid, prophage	Muniesa et al. (2012)
	Enterohaemorrhagic *E. coli* (EHEC)	Gastroenteritis, diarrhoea	Phage-encoded Shiga toxin	*stx*	Phage encoded, plasmid encoded	Pan et al. (2021)
	*Vibrio cholerae*	Gastroenteritis, diarrhoea	Cholera toxin, CTX phage	*ctxA*, *ctxB*	Prophage	Das et al. (2011)
	*Vibrio parahaemolyticus*	Gastroenteritis, diarrhoea	Haemolysin	*trh*	Plasmid	Letchumanan et al. (2014)
	*Helicobacter pylori*	Peptic ulcer disease and gastric adenocarcinoma	Vacuolating toxin	*vacA*	Plasmid	Foegeding et al. (2016)
	*Shigella dysenteriae*	Dysentery	Shiga toxin	*stx1*, *stx2*	Plasmids, insertion sequences, integrons, pathogenicity islands and bacteriophages	Gamage et al. (2004)
	*Yersinia enterocolitica*	Gastrointestinal illness	Siderophore, heat stable enterotoxin	*ystA, ystB, ystC*	Plasmids, ICE	Zheng et al. (2008)
Firmicutes	*Staphylococcus aureus*	Gastrointestinal illness	Enterotoxins, TSST-1, serine protease	*sea*, *seb*, *sec*, *sed*, *see*, *seg*, *seh*, *sei*	Staphylococcal pathogenicity islands (SaPIs), genomic islands, prophages and ICEs	Argudín et al. (2010)
	*Clostridium perfringens*	Gastrointestinal illness, necrotising intestine	Alpha toxin (AT), perfringolysin O toxin (PO)	*cpe*, *plc*, *pfoA*	Plasmids, phages, transposons	Awad et al. (2001)
	*C. difficile*	Inflammation leading to tissue damage	*tcd*B (clostridial cytotoxins) and *cdt*AB (binary toxin)	*tcdA*, *tcdB*	Putative plasmids	Lyras et al. (2009)
	*Clostridium botulinum*	Foodborne intoxication, intestinal infections	Botulinum neurotoxin A, B and E	*neurotoxin A*, *B and E*	Plasmid, prophages Deβ	Franciosa et al. (2009)
	*Enterococcus faecalis*	Erosion of intestinal lining	Cytolysin	*esp*	Pheromone-responsive plasmids, pathogenic islands, prophages	Weaver et al. (2017)
	*Listeria monocytogenes*	Vomiting, stomach disease	Adherence, invasion, enzymes	*hlyA*	Plasmid	den Bakker et al. (2013)
	*Bacillus cereus*	Gastrointestinal illness	Surface antigens	*nhe*, *hbl*	Plasmids, bacteriophages	Sastalla et al. (2013)

## Dynamics of toxin-linked MGEs

As discussed in the above sections of the review, a large number of virulence determinants have been associated with MGEs in important gut pathogens. Though there are several studies and reviews that highlight the importance of MGEs associated with ARGs and their transmission dynamics among the commensals and the pathogens, studies discussing the importance of MGEs associated with virulence genes and their dynamics is sparse. Though HGT between species from different phyla is considered a rare event, it is common within the same species. However, there are also interesting reports of HGTs between even kingdoms where bacterial genes and its protein homologous have resulted in gain of functions in higher order organisms such as fungi, nematodes and eukaryotes (Mayer et al., 2011; Moran and Jarvik 2010). Jaramillo et al. (2015) identified 11 HGT events of toxin genes from bacteria to the plant fungi *Colletotrichum gloeosporioides.* Many toxin genes homologous to subtilisin genes were acquired by the fungi from *Bacillus pumilus.* Further, there are also reports of cross phyla dynamics of toxins with the striking example of aerolysin, a pore forming toxin present in *Aeromonas hydrophila* also identified in many pathogens belonging to the phyla Firmicutes and Proteobacteria (Kennedy et al., 2009). Further, broad host range MGEs have been observed to transcend phyla and mobilise from the commensals to the pathogen isolates (Forster et al., 2022). Forster and his team compared more than 1000 commensal strain genomes belonging to 540 species and more than 45,000 pathogens belonging to 12 species and found more than 64,000 MGE-mediated transfer events between the commensals and the pathogens.

A well-studied transfer of toxin gene within the same bacterial species is the transfer of CT gene from toxigenic *V. cholerae* O1 to environmental non-O1/O139 *V. cholerae* (Choi et al., 2010). *Vibrio* phages that are a common inhabitant of aquatic systems are known to play an important role in the transfer of CT genes (CTX-AB) from a toxigenic strain to a non-toxigenic strain and modulate dynamics and evolution of *V. cholerae.* Transduction experiments were conducted using toxigenic *V. cholerae* O395 and E4 strains to determine the ability of vibrio phages to transfer CTXɸ genes to non-toxigenic strains (Choi et al., 2010). The NetB pore-forming toxin produced by the *C. perfringens* when co-cultured with *netB* negative *C. perfringens* isolates has been identified to acquire the toxin gene through the transfer of the conjugative plasmid pJIR3535 and pNetB-Ne10 (Lacey et al., 2017).

Further, cross species HGT of toxin genes has been demonstrated by Muthukrishnan et al. (2019) through co-culture experiments of *pirAB* positive *V. parahaemolyticus* isolates and *pirAB* negative *Algoriphagus* sp. Strain. The transfer of *pirAB* gene occurs through the conjugative transfer of pVA1 plasmid. The toxin that causes sloughing and degeneration of the hepatopancreas of shrimp has been identified not only in *V. parahaemolyticus* but also in several other *Vibrio* sp. and also non-Vibrios (Dong et al., 2017; Restrepo et al., 2018). Another example of transfer of toxin genes among different species of bacteria is the conjugal plasmid (pVT1) of *V. tapetis* that causes the brown ring disease. The mosaic plasmid is known to contain DNA regions similar to that of *V. vulnificus*, *Photobacterium profundum*, *Listonella anguillarum* and *Shewanella* sp. The dynamics of MGE-linked toxin genes among gut pathogens and non-pathogenic bacteria can happen within the gut and also in the environment. Environmental parameters are known to play a significant impact in the HGT and expression regulation of virulence genes. A biofilm environment is known to increase the rate of HGTs due to the close proximity of the bacterial cells within the biofilm (Gyles and Boerlin, 2013). Additionally, TA systems are also known to contribute in the selection and maintenance of MGEs (Aminov et al., 2011). The TA system comprises a stable toxin present in the chromosome and a labile anti-toxin usually located on plasmids. When the bacterial cells lose the plasmid, the anti-toxin expression ceases and the toxin expression causes cell death. Thus, this two-component system selectively eliminates plasmid-free bacterial cells in a population (Aminov et al., 2011). Hence, understanding the environmental factors affecting the transfer, genetics and dynamics of virulence associated with MGEs will shed light on the evolution of bacteria as well as understand futuristic emerging bacterial pathogens. Further, comprehending broad host MGEs can allow researchers to identify natural and synthetic molecules that can reduce its mobility, preventing virulence and ARG transfer.

## Conclusion

The breakthrough in sequencing technologies has opened the door to examining myriads of microbes inhabiting the human gut. Insights into their genomes have helped in understanding the ecology of different microbes, their functions as well as the dynamics of MGEs linked with various fitness traits. In most of the bacterial enteric pathogens, the reason for the diseased condition is the toxin production, which is encoded by these MGEs. These MGEs include mostly phages, pathogenicity islands, plasmids and transposons. The present review summarises the different MGEs associated with virulence traits of clinically important enteric pathogens. Further studies on the MGEs will pave the way for a better understanding of their bacterial specificity, integration–excision mechanisms as well as inheritance. This understanding can further aid the researchers in designing strategies to prevent the spread of these MGEs that have an important role in alleviating the diseased condition. Further, various strategies like screening of natural and synthetic compounds that can cure the MGEs from bacterial pathogens, thereby making them less virulent and sensitive to existing antibiotics, can be formulated. These strategies, designed to combat the stability of these MGEs, could help to reduce the disease burden and prevent the emergence of antibiotic-resistant mutants in the future.
